# Pharmacogenomic-guided clozapine administration based on HLA-DQB1, HLA-B and SLCO1B3-SLCO1B7 variants: an effectiveness and cost-effectiveness analysis

**DOI:** 10.3389/fphar.2022.1016669

**Published:** 2022-10-14

**Authors:** Kohei Ninomiya, Takeo Saito, Masashi Ikeda, Nakao Iwata, François R. Girardin

**Affiliations:** ^1^ Department of Psychiatry, Fujita Health University School of Medicine, Toyoake, Aichi, Japan; ^2^ Division of Clinical Pharmacology, Department of Laboratory Medicine and Pathology, Lausanne University Hospital, Faculty of Medicine, University of Lausanne, Lausanne, Switzerland

**Keywords:** clozapine, agranulocytosis, granulocytopenia, genotype testing, pharmaco-genomics (PGx), pharmaco-economics, human leukocyte antigen (HLA), schizophrenia

## Abstract

The identification of pharmacogenetic factors that increase the susceptibility to clozapine-induced agranulocytosis or granulocytopenia (CIAG) has received increasing interest. The SLCO1B3-SCLO1B7 variant (rs149104283) and single amino acid changes in human leukocyte antigen (HLA) HLA-DQB1 (126Q) and HLA-B (158T) were associated with an increased risk of CIAG. In this study, we evaluated the effectiveness and cost-effectiveness of adding the SLCO1B3-SCLO1B7 to HLA variants as a new pharmacogenomic (PGx) approach and explored the evolution of a cohort of schizophrenic patients taking long-term clozapine as a third-line antipsychotic medication. The decision model included probabilistic and deterministic sensitivity analyses to assess the expected costs and quality-adjusted life-years (QALYs). The current monitoring scheme was compared with the PGx-guided strategy, where all patients underwent pre-emptively a genetic test before taking clozapine, over 10 years. By adding the SLCO1B3-SCLO1B7 variant into HLA variants, CIAG sensitivity increased from 36.0% to 43.0%, the specificity decreased from 89.0% to 86.9%, and the probability of cost-effectiveness improved from 74.1% to 87.8%. The incremental cost-effectiveness ratio was £16,215 per QALY and remained below the conventional decision threshold (£30,000 or US$50,000 per QALY). Therefore, the SLCO1B3-SCLO1B7 variant, as an additional risk allele to HLA variants, increases preemptive test sensitivity and improves the effectiveness and cost-effectiveness of PGx-guided clozapine administration.

## 1 Introduction

In most Western and Asian countries, approximately 1%–3% of patients taking clozapine (CLZ) experience severe neutropenia that occurs within several weeks of treatment (J M Alvir et al., 1993). However, drug-induced granulocytopenia and agranulocytosis are distinct phenotypes with different etiologies, risk factors, evolution dynamics, and distinct outcomes. CLZ-induced neutropenia usually occurs after 1–2 weeks of exposure and is more frequent in Africans with low baseline leukocyte count, and the degree of neutropenia depends on the dose and duration. CLZ-induced agranulocytosis (CIA) typically becomes obvious 2–8 weeks after the initiation of therapy, has large idiosyncratic and genetic components, and is more frequent in Asians, females, and the elderly (Flanagan and Dunk, 2008); a low baseline leukocyte count was not associated with CIA.

A genome-wide association study detected that the human leukocyte antigen (HLA) region (single amino acid changes in HLA-DQB1 (126Q) and HLA-B (158T)) (Goldstein et al., 2014) and SLCO1B3-SCLO1B7 (rs149104283) (Legge et al., 2017) were associated with genetic susceptibility to CIA in European ancestry. The association of HLA and *SLCO* alleles with an increased risk of agranulocytosis suggests an immune-mediated mechanism combined with an altered function of drug influx transporter that could affect myeloid precursors translating into CIAG (CIA + CLZ-induced granulocytopenia (CIG)). Influx transporter polymorphisms with altered activity have also been implicated in further adverse reactions of simvastatin-induced myopathy (E. Link et al., 2008) and docetaxel-induced neutropenia (Chew et al., 2012). The SLCO1B3-SCLO1B7 (rs149104283) variant is an intronic single-nucleotide polymorphism to transcripts of the hepatic transporter genes SLCO1B3 and SCLO1B7 (Legge et al., 2017), which could at least partly explain the pharmacokinetic origin of neutropenia.

Pharmacogenomic (PGx) profiles and information could be integrated into clinical settings to reduce CLZ discontinuation for hematological concerns and to improve mental health outcomes. This is particularly topical because current strategies for monitoring leukocyte count in patients taking CLZ remain based on divergent national schemes that are not cost-effective (Girardin et al., 2014). For patients taking CLZ in the US, the UK, Switzerland, and Japan, HLA genotype-guided blood monitoring appeared to be a cost-effective strategy compared with either absolute neutrophil count monitoring or CLZ substitution by other less effective antipsychotics (Ninomiya et al., 2021); (Girardin et al., 2019).

In this study, we investigated whether adding the SLCO1B3-SCLO1B7 variants to the HLA PGx-guided approach is efficient to leverage the performance in predicting CIAG development in patients taking CLZ as a third-line antipsychotic medication.

## 2 Materials and methods

### 2.1 Decision analytic model and PGx-guided strategy

**FIGURE 1:**
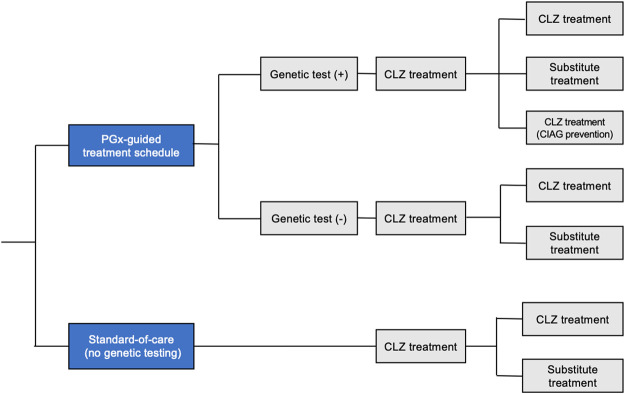
Decision tree schematic. The “Standard-of-care (no genetic testing)” compared with the “PGx-guided treatment schedule.” CIAG, clozapine-induced agranulocytosis/granulocytopenia.

We evaluated the effectiveness and cost-effectiveness of adding the SLCO1B3-SCLO1B7 variant (rs149104283) to HLA variants as a new PGx approach in patients taking long-term CLZ (Figure 1).

To compare the results with the current absolute neutrophil count monitoring schemes conducted in the UK as base-case, we used a decision model to explore the evolution of a cohort of adult men and women who received CLZ as a third-line antipsychotic medication.

To assess the model and parameter uncertainty and to test the robustness of findings based on increased PGx test sensitivity (after incorporating the SLCO1B3-SCLO1B7 variant), the Markov model included deterministic and probabilistic sensitivity analyses to calculate the expected costs and quality-adjusted life-years (QALYs) over 10 years. We compared current monitoring schemes with a “PGx-guided strategy,” where all patients underwent pre-emptively a genetic test before taking CLZ.

Derived from previous analyses, the decision model was based on two mutually exclusive strategies, namely, the “PGx schedule” and the “common schedule without genetic testing as a standard-of-care” (Ninomiya et al., 2021).

PGx-guided treatment schedule: In this scenario, all patients underwent genetic testing and were divided into two groups based on the presence of risk alleles. The risk of developing CIAG was higher in patients with risk alleles than in those without risk alleles. However, due to the low positive predictive value of this genetic testing (approximately 10%), not all patients with risk alleles developed CIAG. Furthermore, antipsychotic substitution was less efficient in achieving quality of life than clozapine treatment. Thus, having risk alleles does not indicate discontinuation of the CLZ treatment in clinical setting. Therefore, we set up a scenario analysis from the base-case where patients with risk alleles receive clozapine, and it is expected that *a priori* information of the specific patients with genetic risk would alert the psychiatrists’ attitude. The overall CIAG onset rate should be reduced with the psychiatrists’ awareness of the potential risk and sensitivity to CIAG in these patients. Genetic variants (rs149104283 and HLA-DQB1 (126Q) and HLA-B (158T)) will be considered as additional risk alleles, and prescribers should apply stringent blood monitoring or early “temporary cessation” of CLZ treatment to avoid the “complete discontinuation” of CLZ treatment, which are expected to reduce the overall CIAG onset rate. The CIAG prevention rate was set at 30%, based on previous findings (Ninomiya et al., 2021). Blood monitoring was conducted weekly in the first 18 weeks of CLZ treatment and then every 2 weeks, in accordance with the Clozaril Patient Monitoring Service (CPMS) protocol. If CIAG occurred, CLZ treatment was discontinued and switched to substitute antipsychotic treatment.

In the vast majority of cases, patients did not harbor risk alleles and follow the “Standard-of-care (no genetic testing)” (see below).

Standard-of-care (no genetic testing): This corresponds to the current monitoring schedule used in Japan and most Western countries.

In brief, during 18 weeks of CLZ treatment, weekly blood monitoring is performed, and after that, blood monitoring occurs every 2 weeks. However, if the white blood cell count (WBC) or absolute neutrophil count (ANC) decreases to <3,000/mm^3^ or 1,500/mm^3^, CLZ treatment should be discontinued (rechallenging for patients with CIAG is also prohibited unless the CPMS committee gives permission based on the clinical course) at that moment. The relaxation of the criteria for the entry to the UK CLZ central non-rechallenge database has been modeled recently (Oloyede et al., 2022).

In these models, the possibility of CLZ discontinuation due to WBC cutoff (<3,000/mm^3^) was not considered because the definition of “WBC count” does not usually indicate CLZ discontinuation (in such cases, the ANC usually decreased to 1,500/mm^3^) (Myles et al., 2018).

All patients received CLZ treatment and if CIAG occurred, CLZ treatment was discontinued and switched to substitutional treatment.

### 2.2 Population, model structure, and parameters

The target population was identical to that reported previously (Girardin et al., 2019): adult men and women from the UK with treatment resistance schizophrenia who are eligible for CLZ treatment. We used a Markov model for assessing the transition probability (cycle length: 1 month). The model incorporated the health status of the patients to reflect that they received either CLZ or substitute antipsychotic treatments.

Key driving parameters were previously identified: 1) CIAG prevalence (3.43%) (Freeman et al., 2016); 2) cost of treatment for CIAG (£469.48) (Jin et al., 2019); 3) cost of CLZ/day (£1.23) (National Health Service in the UK, 2019)^,^ (Heeg et al., 2008); 4) cost of substitute/day (£5.11), which was calculated by weighting the cost × the percentage of the first-line drugs used in the UK (risperidone: 21.5%, aripiprazole: 10.8%, olanzapine: 19.7%, quetiapine: 42.8%, and amisulpride: 5.2%), because one type of second-generation antipsychotic is commonly prescribed for schizophrenia in the UK (National Health Service in the UK, 2019)^,^ (Patel et al., 2014); 5) cost of genetic tests (£110: we assumed £100 for HLA typing and £10 for rs149104283 genotyping); 6) cost of regular blood test/month (£10.6); 7) utility for patients undergoing CLZ treatment (0.693), which was estimated from the report on the basis of the EQ-5D index score (Sullivan and Ghushchyan, 2006); 8) utility for patients undergoing substitute treatment (0.560), which was estimated from the report on use of standard gamble, rating scales, and paired comparison questions (Revicki et al., 1996); and 9) the CIAG prevention rate [30% (Ninomiya et al., 2021)] (Table 1). The costs related to medical fees were calculated according to the direct Medical Care Expenditure based on the National Health Service in the UK (April 2019) (National Institute of Public Health, 2019).

**TABLE 1:** Input parameters.

Parameter	Mean	Probabilistic sensitivity analysis	Type of distribution	Distribution parameter	References
CIAG prevalence	3.43%	NO			Freeman et al. (2016)
Cost of treatment CIAG £	469.48	YES	Gamma	alpha:4 lambda:8.5E-3	Jin et al. (2019)
Cost of CLZ/day £	1.23	YES	Gamma	alpha:37.8 lambda:30.75	National Health Service in the UK (2019), Heeg et al. (2008)
Cost of substitute/day £	5.11	YES	Gamma	alpha:104.4 lambda:20.44	National Health Service in the UK (2019), Patel et al. (2014)
Cost of genetic test £	110	NO			
Cost of regular blood test/month £	10.6	NO			
Utility for patients undergoing clozapine treatment	0.693	YES	Beta	alpha:575 beta:255	Girardin et al. (2019)
Utility for patients undergoing substitute treatment	0.560	YES	Beta	alpha:86 beta:67	Girardin et al. (2019)
CIAG prevention rate	30%	YES	Beta	alpha:24.9 beta:58.1	Ninomiya et al. (2021)
Sensitivity^a^	43.0%	YES	Beta	alpha:169.13 beta:223.87	Legge et al. (2017), Goldstein et al. (2014)
Specificity^a^	86.9%	YES	Beta	alpha:15531.77 beta:2342.23	Legge et al. (2017), Goldstein et al. (2014)

^a^ Based on the combined risk of HLA-DQB1 (126Q) and HLA-B (158T) and rs149104283.

CIAG, Clozapine-induced agranylocytosis/granulocytopenia. CLZ, clozapine.

Aggregated sensitivity and specificity of allelic variants [rs149104283 (Legge et al., 2017), HLA-DQB1 (126Q), and HLA-B (158T) (Goldstein et al., 2014)] were calculated as follows:

Sensitivity=1−(1−Sensitivity1)×(1−Sensitivity2)=1−(1−0.360)×(1−0.109)=0.430

Sensitivity1=sensitivity of HLA‐DQB1(126Q) and HLA‐B(158T)=0.36

Sensitivity2=sensitivity of rs149104283=0.109

Specificity=Specificity1×Specificity2=0.890 × 0.976=0.869

Specificity1=specificity of HLA‐DQB1(126Q) and HLA‐B (158T)=0.890

Specificity2=specificity of rs149104283=0.976

The outcomes included the mean cost-per-patient and QALY-per-patient for calculating the incremental cost-effectiveness ratio (ICER) for 10 years.

Probabilistic sensitivity analysis was performed using Monte Carlo simulations by varying input parameters (95% confidence intervals or clinically reasonable ranges). We set the number of simulations to 100,000 based on randomly assigned parameters. We obtained the costs and QALY values for both strategies and calculated the ICER based on the following formula:

$$\frac{Cost\;\left(PGx‐guided\;treatment\;schedule\right)-Cost\;\left(Standard‐of‐care\right)}{QALY\left(PGx‐guided\;treatment\;schedule\right)-QALY\;\left(Standard‐of‐care\right)}$$

The discount rate of 3.5% was applied to the costs and QALYs. The cost-per-QALY thresholds were set at £30,000, as recommended in the UK guidelines (National Institute of Public Health, 2019).

The sample size of the combined CLOZUK and CIAG Consortium (CIAC) (229 cases and 13,553 controls) had 80% power to detect a relative risk (RR) > 3 with minor allele frequency (MAF) > 0.10 at *p* < 5 × 10^–8^. We defined “undetected risk variants” (RR ≤3; MAF, ≤0.10) that can be a detectable risk by increasing the sample size, with various allele frequencies and relative risks. To estimate the minimum number of cases required, we used the Genetic Association Study Power Calculator (Goncalo, 2017).

The sensitivity and specificity derived from MAF and relative risk for “undetected risk variants” and those from HLA-DQB1 (126Q), HLA-B (158T), and SLCO1B3-SLCO1B7 yielded calibrated sensitivity and specificity estimates. We set the cost for additional genetic tests ranging from £110 to £130 to reflect the probability.

All CEAs followed the Guideline for Preparing Cost-Effectiveness Evaluation to Consolidated Health Economic Evaluation Reporting Standards (CHEERS) guideline (Husereau et al., 2013).

TreeAgePro® (2019 version, TreeAge Software Inc. MA, United States) was used for the decision model, sensitivity analyses, and simulations.

## 3 Results

Our findings indicated that if the SLCO1B3-SCLO1B7 variant was added to HLA variants, CIAG sensitivity increased from 36.0% to 43.0%, and the specificity decreased from 89.0% to 86.9%. Based on the CIAG incidence of 3.43% and test sensitivity of 0.43, the number of patients needed for genotyping was estimated as follows: (100/(3.43 × 0.43). Overall, 68 screened patients were needed to prevent one case of CIAG and 232 patients were needed to prevent one case of severe CIA (<500/mm^3^). These estimates approximate previous estimations with agranulocytosis prevalence but with lower single HLA genotyping sensitivity (Girardin et al., 2019).

From a pharmaco-economic perspective, the probability of cost-effectiveness improved from 74.1% to 87.8%, and the ICER was £16,215 per QALY, indicating that it remained well below the conventional decision threshold (£30,000 or US$50,000 per QALY).

**FIGURE 2:**
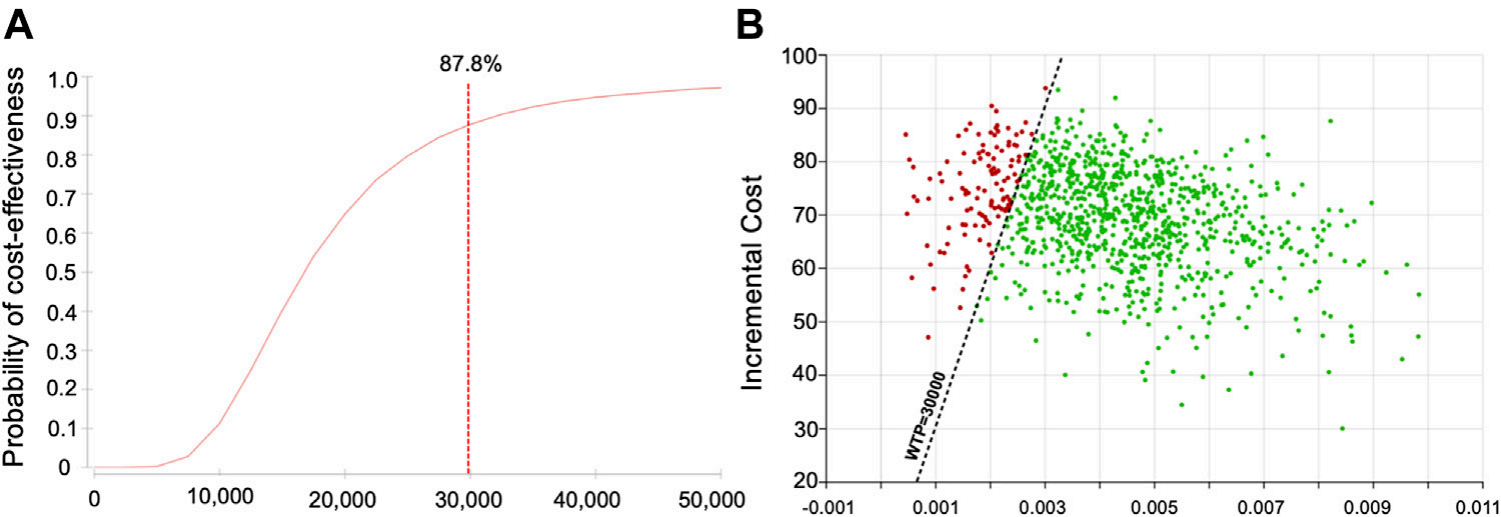
Results of probability sensitivity analysis [HLA-DQB1 (126Q), HLA-B (158T) and rs149104283] **(A)** Cost-effectiveness acceptability curve **(B)** Scatter plot for incremental cost and effectiveness: green dots indicate ICERs within willing to pay (WTP) threshold and red dots indicate out of the threshold.

**FIGURE 3:**
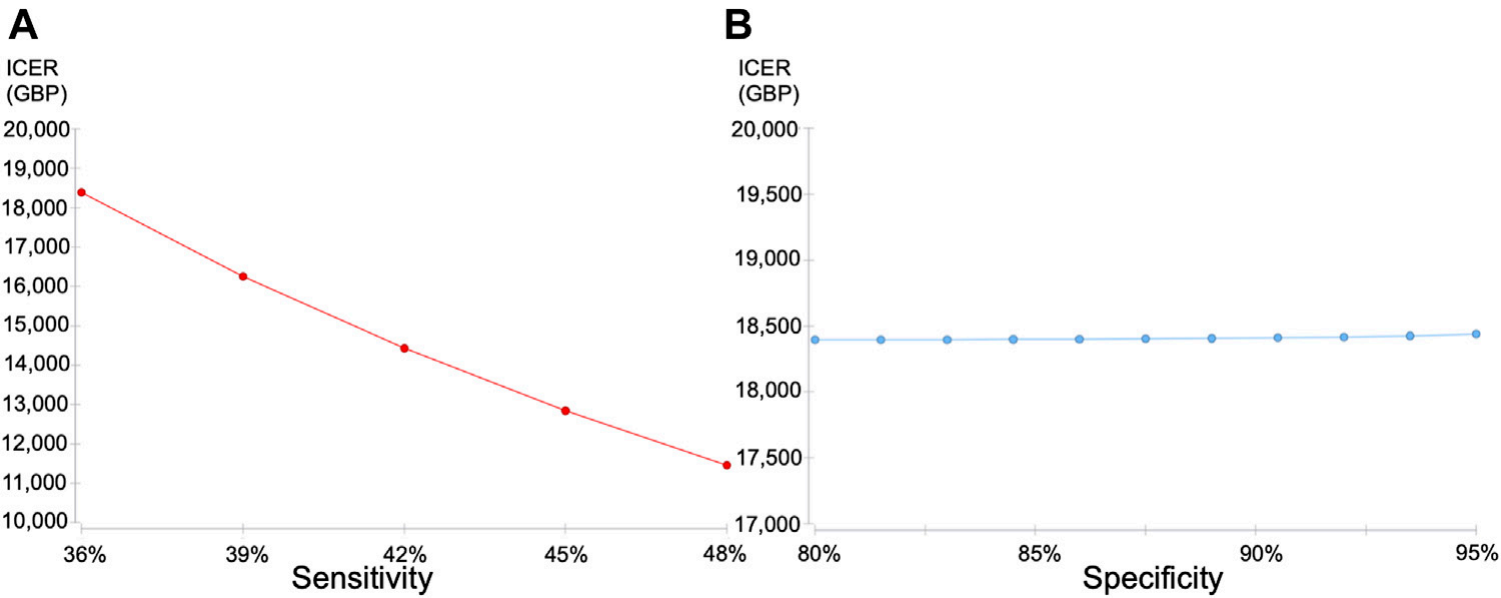
Results of one-way sensitivity analysis **(A)** Sensitivity analysis: varying “sensitivities” **(B)** Sensitivity analysis: varying “specificities”, ICER incremental cost-effectiveness ratio.

Hence, the PGx-guided schedule appeared as an acceptable alternative to the current blood monitoring schedule (standard-of-care) (Figure 2). To comprehend the effects of specificity reduction, we conducted one-way sensitivity analysis for sensitivity and specificity, respectively (Figure 3). Better ICERs were obtained when we increased the sensitivity; however, the ICERs did not change when various specificities were examined.

We considered a further scenario where the “undetected risk variants” have a relative risk of three and an allele frequency of 5%: we found increased CIAG sensitivity from 43.0% to 56.8% and the specificity decreased from 86.9% to 78.9% by adding the “undetected risk variants” into the HLA and SLCO1B3-SCLO1B7 (rs149104283) variants. Under these hypothetical conditions, the probabilistic estimate of the total cost was £4,278 and that for QALYs was 5.83134 for the PGx-guided strategy. The expected ICER was calculated at £11,819, and the probability of cost-effectiveness was 94.8%, indicating that even under the assumption of further undetected risk alleles, the PGx-guided strategy remained within the acceptable range of cost-effectiveness (Supplementary Figure S1).

Further results and findings associated with an increased relative risk and MAF scenario are provided in Table 2. The number of required cases will be increased obviously if the relative risk and allele frequency decrease. However, it is of note that the ICERs for any relative risk will be smaller than the ICER for the base-case (HLA + SLCO1B3-SLCO1B7 variants), if the MAF is greater than 2.5%.

**TABLE 2:** The number of cases to obtain 80% power under various relative risk and allele frequency of SNPs, and ICER for each model.

Relative risk	Allele frequency	10%	7.5%	5%	2.5%	1%
3.0	Case	230	270	360	640	1,510
	ICER (£/QALY)	8657.73	9959.48	11759.02	14300.20	16539.54
2.5	Case	330	390	530	950	2290
	ICER (£/QALY)	9368.75	10665.39	12391.99	14831.65	16829.12
2.0	Case	570	690	940	1740	4470
	ICER (£/QALY)	10246.28	11518.55	13155.14	15413.00	17142.65

ICER, incremental cost-effectiveness ratio.

## 4 Discussion

To the best of our knowledge, this study is the first comparative cost-effectiveness analysis using two alternative strategies based on pharmacogenomic testing of the SLCO1B3-SCLO1B7 variant in addition to single HLA alleles. Furthermore, as future scenario analysis, if more risk variants will be detected and integrated into this model, even lower ICERs can be obtained as a collateral effect of incorporating additional PGx results.

CIAG or CIA alone impacted the number needed to genotype because the prevalence rates were significantly different (1% vs 3.43%). The pharmacoeconomic findings indicated that the extended PGx approach yielded an ICER of £16,215 per QALY, which remained well below the willing to pay threshold for one additional QALY (i.e., <£30,000/QALY or < US$50,000 per QALY). Furthermore, we used a probabilistic framework to explore joint parameter uncertainty and whether parameter variability is translated into outcome variability to capture the costs and consequences, as shown on the cost-effectiveness acceptability curve (Figure 2): incorporating the SLCO1B3-SCLO1B7 variant to HLA variants improved the probability of cost-effectiveness from 74.1% to 87.8%.

In this model, the genetic test sensitivity, which improved from 36.0% to 43.0%, largely contributed to cost-effectiveness improvement, even though the test specificity decreased marginally from 89.0% to 86.9%. This is supported by our probabilistic sensitivity analyses.

This result also indicates that increasing the “risk” variants improves PGx test sensitivity and cost-effectiveness. As mentioned in Table 2, even small risks have a significant impact on ICER. However, sensitivity limits exist, which largely depend on the novel PGx evidence.

Psychiatrists’ prior information for identifying the risk of CIAG could facilitate the use of CLZ, which has economic and clinical benefits for managing patients with treatment-resistant psychosis, such as schizophrenia, or neurodegenerative diseases with extrapyramidal syndromes. Even though the translation processes with clinical implementation of PGx, including proof of effectiveness and cost-effectiveness, have been emphasized (Swen et al., 2007), these findings expand the knowledge for optimizing resource allocation and wisely choosing campaigns (Cassel and Guest, 2012). Moreover, intensive blood monitoring requirements associated with CLZ prescription could delay drug initiation and impede patient recovery. Regulatory agencies, including the Food and Drug Administration, revised the requirements for blood monitoring and dispensing of CLZ with updated risk evaluation and mitigation strategies. Revisions include prescribing CLZ for patients with benign neutropenia and using algorithms and artificial intelligence-based tools for patients who benefited from atypical antipsychotic medications but had CIAG. Recently, a modeling study indicated that the CLZ rechallenging success after patients’ hematological parameters falls below particular thresholds, namely, the CLZ central non-rechallenge database (CNRD); the success rates were similar between individuals who did not meet the CNRD registration criteria and those who did meet those criteria (Oloyede et al., 2022).

The limitations of the study, considering preemptive implementation of PGx-guided CLZ prescription, are undoubtedly related to assumptions regarding the consequences of limited test sensitivity. As we considered the SLCO1B3-SCLO1B7 variant, genotyping performance remains the driving parameter for generalizable genetic testing. It is hardly advisable to stop blood monitoring without formal pilot studies and transition periods. The second limitation is the restricted long-term data in registries: this analysis could not extend beyond 10 years of observation without strong assumptions. Eventually, because we used a third-party payer perspective, we could incorporate neither intangible costs, such as productivity loss related to premature death, nor unintended follow-up benefit.

The study strengths are the key parameters derived from a large CIAG consortium. The decision analytical framework was built using various deterministic and probabilistic sensitivity analyses, with conservative estimates and scenarios, to provide robust results. The costs were derived from hospital statistics and diagnosis-related group rates derived from hospital admissions.

We concluded that adding risk alleles to HLA variants would increase test sensitivity and improve the effectiveness and cost-effectiveness of PGx-guided CLZ administration.

## Data Availability

The original contributions presented in the study are included in the article/Supplementary Material, further inquiries can be directed to the corresponding author.

## Supplementary material

The Supplementary Material for this article can be found online at: https://www.frontiersin.org/articles/10.3389/fphar.2022.1016669/full#supplementary-material

SUPPLEMENTARY FIGURE S1 Results of probability sensitivity analysis [HLA-DQB1 (126Q), HLA-B (158T), rs149104283 and “undetected risk variants” (relative risk = 3, minor allele frequency = 5%)] **(A)** cost-effectiveness acceptability curve **(B)** Scatter plot for incremental cost and effectiveness: green dots indicate ICERs within willing to pay (WTP) threshold and red dots indicate out of the threshold.
