# Supplementary figures and images for: Pharmacogenomic-guided clozapine administration based on HLA-DQB1, HLA-B and SLCO1B3-SLCO1B7 variants: an effectiveness and cost-effectiveness analysis
Source: Front Pharmacol. 2022 Oct 14;13:1016669. doi: 10.3389/fphar.2022.1016669 (PMC9614368; doi:10.3389/fphar.2022.1016669)

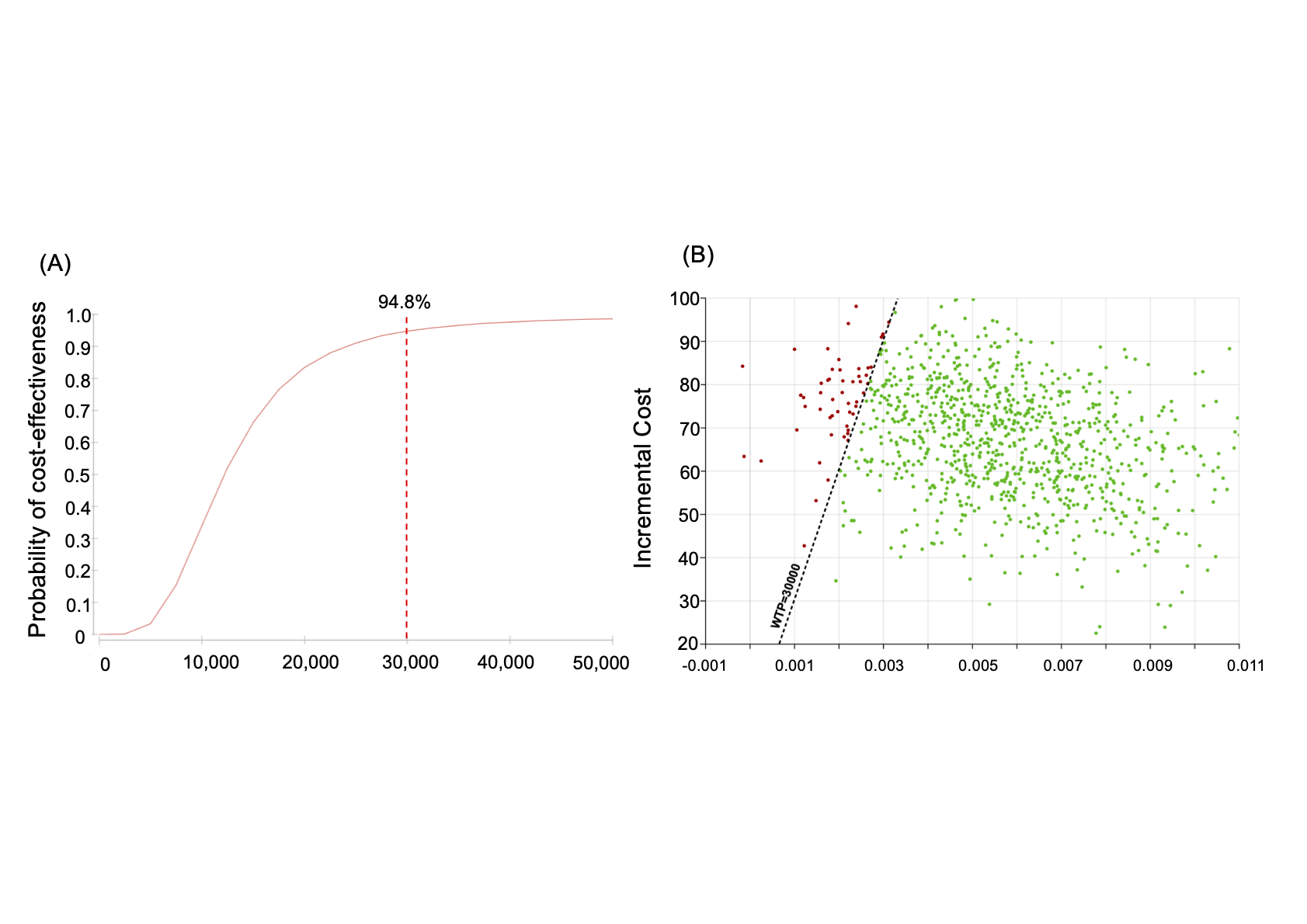

Supplement: Supplementary file 1 [file Image1.tiff]
